# Endogenous stimulation is responsible for the high frequency of IL-17A-producing neutrophils in patients with rheumatoid arthritis

**DOI:** 10.1186/s13223-019-0359-9

**Published:** 2019-08-01

**Authors:** Maria Gonzalez-Orozco, Rosa E. Barbosa-Cobos, Paola Santana-Sanchez, Lizbeth Becerril-Mendoza, Leonardo Limon-Camacho, Ana I. Juarez-Estrada, Gustavo E. Lugo-Zamudio, Jose Moreno-Rodriguez, Vianney Ortiz-Navarrete

**Affiliations:** 1 Departamento de Biomedicina Molecular, Centro de Investigación y de Estudios Avanzados del Instituto Politécnico Nacional, Av IPN 2508, 07360 Mexico City, Mexico; 2 Servicio de Reumatología, Hospital Juarez de Mexico, Av. IPN 5160, 07760 Mexico City, Mexico; 3 Servicio de Reumatología, Hospital Central Norte, Pemex, Campo Matillas 52, 02720 Mexico City, Mexico; 4 Direccion de Enseñanza e Investigacion, Hospital Juarez de Mexico, Av. IPN 5160, 07760 Mexico City, Mexico

**Keywords:** Rheumatoid arthritis, Neutrophils, IL-17, DAS-28, Th17

## Abstract

**Background:**

Neutrophils play an important role in the pathogenesis of rheumatoid arthritis (RA). It has recently been reported that in addition to T helper (Th) 17 cells, other cells, including neutrophils, produce IL-17A, an important inflammatory cytokine involved in the pathogenesis of RA. The purpose of this study was to examine the presence of interleukin 17A-producing neutrophils in patients with RA.

**Methods:**

We performed a cross-sectional study including 106 patients with RA and 56 healthy individuals. Whole peripheral blood cells were analyzed by flow cytometry to identify CD66b+ CD177+ IL-17A+ neutrophils and CD3+ CD4+ IL-17A+ T cells. Serum levels of IL-17A and IL-6 were measured by means of cytometry bead array (CBA). In purified neutrophils, mRNA levels of IL-17 and RORγ were measured by RT-PCR. In addition, purified neutrophils from patients and healthy controls were stimulated with the cytokines IL-6 and IL-23 to evaluate differences in their capacity to produce IL-17A.

**Results:**

Neutrophils from RA patients expressed IL-17 and RORγ mRNA. Consequently, these cells also expressed IL-17A. Serum IL-17A levels but not Th17 cell numbers were increased in RA patients. Neutrophils positive for cytoplasmic IL-17A were more abundant in patients with RA (mean 1.2 ± 3.18%) than in healthy individuals (mean 0.07 ± 0.1%) (*p *< 0.0001). Although increased IL-17A+ neutrophil numbers were present in RA patients regardless of disease activity (mean 6.5 ± 5.14%), they were more frequent in patients with a more recent diagnosis (mean time after disease onset 3.5 ± 4.24 years). IL-6 and IL-23 induced the expression of RORγ but failed to induce IL-17A expression by neutrophils from RA patients and healthy individuals after a 3 h stimulation.

**Conclusion:**

IL-17A-producing neutrophils are increased in some RA patients, which are not related to disease activity but have an increased frequency in patients with recent-onset disease. This finding suggests that IL-17A-producing neutrophils play an early role in the development of RA.

**Electronic supplementary material:**

The online version of this article (10.1186/s13223-019-0359-9) contains supplementary material, which is available to authorized users.

## Background

RA is a systemic and chronic inflammatory autoimmune that affects 1–1.5% of the population worldwide [1]. The inflammatory process behind RA is associated with an increased production of inflammatory cytokines and chemokines. Among others, tumor necrosis factor (TNF) and interleukin (IL)-6 appear to play a major role in the pathogenesis of tissue damage in RA, as evidenced by a decrease of disease activity after blocking of these cytokines [2]. Other cytokines involved in the pathogenesis of RA include interferon (IFN)-γ and IL-17, which are the major effector cytokines of the Th1 and Th17 subsets of CD4+ T lymphocytes, respectively [3, 4]. Of these, IL-17 (mostly IL-17A) has been the subject of interest over the past two decades. Thus, IL-17A levels are increased in the synovial fluid and serum [5–7], and Th17 cells are enriched in the synovial membrane of RA patients [6]. Moreover, the K/BxN mouse model of arthritis supports the role of IL-17 in the pathogenesis of joint inflammation [8]. Although preliminary clinical trials targeting IL-17A in RA have shown some clinical benefits, these positive results have yet to be confirmed in large-scale clinical trials [4, 9, 10].

In addition to Th17 cells, mast cells and mononuclear cells are present in the synovium of RA patients. Additionally, human and mouse neutrophils are capable of producing IL-17A [11–21], but no evidence for these cells has been linked to RA. In the case of IL-17A-producing neutrophils, they are increased in the peripheral blood of patients with asthma who are allergic to fungus [22], as well as in the plaques of psoriasis patients [23], and in the joints of patients with ankylosing spondylitis but not in those of patients with osteoarthritis [24]. In RA patients, neutrophils are known to accumulate in the synovial fluid and, to a lesser extent, in the synovial tissue [25]. In mouse models of RA, IL-17A+ neutrophils appear to be important effectors of disease pathogenesis and tissue damage [8]. In addition, peripheral blood neutrophils from patients with RA are functionally different from those obtained from healthy people, with remarkable differences in gene and protein expression, such as that of TNF and myeloblastin. Moreover, RA neutrophils are primed for ROS production [1]. We hypothesized that IL-17A-producing neutrophils would be present in the peripheral blood of patients with RA but not in that of healthy counterparts. To evaluate this hypothesis, we analyzed a cohort of 106 patients with RA and 56 healthy individuals. We found a high frequency of IL-17A-producing neutrophils in patients with RA but not in healthy controls. This result suggests that neutrophils are an important source of IL-17A during RA pathogenesis.

## Methods

### Patients and control subjects

We enrolled 106 RA patients at the Rheumatology Department outpatient clinics of the Hospital Juárez de México (HJM) who fulfilled the ACR/EULAR 2010 criteria for the classification of RA [26, 27]. Briefly, these criteria were a confirmed presence of synovitis in at least 1 joint, an absence of an alternative explanation for synovitis, and a total score of 6 of a possible 10 from the individual scores in (A) number and site of involved joints, (B) serologic abnormality (positive anti-CCP), (C) elevated acute-phase reactants, and (D) duration of symptoms. The enrolled RA patients had a mean disease duration of 9.4 ± 0.81 years (0.5–42 years) since their initial clinical symptoms; thus, all RA patients were receiving drug treatment. Demographic and clinical characteristics were obtained by an interview on the day of sample collection for laboratory studies. The data collected included age, sex, and age of disease onset. Physical examination included number of tender joint, number of swollen joint count, and the disease activity score calculated for 28 joints using C reactive protein (DAS28-CRP).

CRP was determined by means of CardioPhase *hs*CRP, (Siemens Healthcare Diagnostic, Marburg, Germany). Immunologic tests included rheumatoid factor (RF) determined by means of Tina-quant FR II (Roche Diagnostic, Indianapolis, IN, USA) and anti-citrullinated protein antibody levels (ACPAs) determined by ARCHITECT Anti-CCP (Abbott Laboratories, Wiesbaden, Germany). The healthy controls (HC) were 56 age- and sex-matched individuals.

Patients were being treated with different combinations of synthetic disease-modifying antirheumatic drugs (DMARDs) (methotrexate, leflunomide, sulfasalazine or/and hydroxychloroquine) as DMARD monotherapy (34.7%), double DMARD therapy (39.1%), triple DMARD therapy (8.7%), or prednisone plus at least one DMARD (16.3%) (Additional file 1: Table S1). One patient was not taking any medication at the time of sample collection.

The study followed the guidelines of the Declaration of Helsinki and was approved by the Ethics Committee of the HJM. Patients and controls signed informed consent documentation to provide peripheral blood by a single needle puncture, and none of them had any sign of infection.

### Serum collection

Peripheral blood was collected in a tube without anticoagulant and left undisturbed for approximately 30 min. The clot was removed by centrifugation, and sera was aliquoted and kept frozen until cytokine determination.

### PBMC staining

PBMCs were isolated from heparinized whole blood cells (HWB), carefully layered on Ficoll-Paque Plus (GE Healthcare, Uppsala, Sweden) and centrifuged for 30 min. Lymphocyte and monocyte layers were collected and stained using anti-CD3-FITC, anti-CD4-PE and anti-CD8-Pacific Blue antibodies (Biolegend, San Diego CA). Cells were permeabilized using Perm2 (BD Biosciences, San Jose, CA, USA) according to the manufacturer’s recommendations and then stained for detection of intracellular IL-17A with an anti-IL-17A-PercP-Cy5.5 antibody (Biolegend). Samples were analyzed by flow cytometry.

### Peripheral blood staining

HWB were initially stained with antibodies for the neutrophil surface markers CD177-FITC and CD66B-PE (Biolegend, San Diego CA) or with isotype controls for 20 min at 4 °C. Afterward, cells were permeabilized for 15 min with FACS Perm2 solution (BD Biosciences, San Jose, CA, USA) according to the manufacturer’s recommendations. Then, samples were stained with anti-IL-17A-PercP-Cy5.5 or anti-IL-17A-PE (Biolegend) and analyzed using a Fortessa LSR cytometer (BD Biosciences).

### Neutrophil purification and RT-PCR

Erythrocytes were removed from HWB following dextran sedimentation, and neutrophils were isolated by a Ficoll-Hypaque gradient from the leukocyte (nonmononuclear) fraction. Neutrophil purity was 96%, as defined by staining with an anti-CD66b antibody; cell viability was 98% immediately after purification and 90% after 16 h in culture, as defined by the trypan blue exclusion test. RNA was isolated using a Direct-zol kit (Zymo Research Corp, Irvine CA) according to the manufacturer’s directions. The RNA was used to generate cDNA using SuperScript First Strand (Invitrogen, Carlsbad CA) according to the manufacturer’s directions, using the primers for IL-17A: forward TCCCACGAAATCCAGGATGC and reverse GGATGTTCAGGTTGACCATCAC, for RORγ: forward CCTGGGCTCCTCGCCTGACC and reverse TCTCTCTGCCCTCAGCCTTGCC and for GAPDH: forward GTCTCCTCTGACTTCAACAGCG and reverse ACCACCCTGTTGCTGTAGCCAA. RT-PCR was performed using Sso advanced Universal SYBR Green Supermix (Bio-Rad, Hercules CA).

### Neutrophil stimulation and IL-17A quantification

Neutrophils were isolated from healthy controls and RA patients as described above, and 2 × 10^6^ cells were stimulated for 3 h with IL-6 (20 μg/ml) and IL-23 (2 μg/ml) as described [18]; during the last 2 h of stimulation, cells were cultured in the presence of brefeldin A (Sigma Aldrich, Saint Louis, Missouri). After stimulation, cells were collected and stained for flow cytometry as described before. Supernatants were collected after 16 h of stimulation, and IL-17A was quantified by BD cytometry bead array (CBA) following the manufacturer’s instructions.

### Statistics

Statistical analysis was performed using GraphPad Prism Software v5 (GraphPad Prism Software Inc.). All data were analyzed for the D’Agostino & Pearson omnibus normality test. The definition of statistical significance was set at p < 0.05 by means of the nonparametric Mann–Whitney test and by the nonparametric ANOVA Kruskal–Wallis test when more than two groups were examined. Correlation analysis was performed using Spearman’s nonparametric method.

## Results

### Patients and control characteristics

Demographic characteristics and ACPA, RF, CRP and ESR data for the 106 patients and 56 healthy controls are shown in Table 1. Eighty-eight percent of patients were women, and twelve percent were men. The mean DAS28-CRP was 3.2 ± 1.27. Among all patients, 13.8% had at least one first-degree relative with autoimmune disease. The percentage of RF-seropositive patients was 86.1% (> 60 U/ml). The extra-articular manifestations secondary to RA were observed, as follows: 79.4% of the patients had kerato-conjunctivitis sicca, 16% of the patients had current rheumatoid nodules, 1.8% had Raynaud’s phenomenon and 0.9% of patients had cutaneous vasculitis. Of the healthy controls, 65% were women, and 35% were men. None of them had first-degree relatives with autoimmune disease.

**Table 1:** Characteristics of study subjects

	RA patients	Healthy controls
Sex women/men	93/13	39/17
Age (year) (mean ± SEM)	50.7 ± 1.2	43.2 ± 1.9
ACPA (UI/l) (mean ± SD)	322.2 ± 282.1	ND
RF (U/ml) (mean ± SD)	431.2 ± 701.98	ND
ESR mm/h (mean ± SD)	32.4 ± 13.25	19.2 ± 11.36
CRP mg/dl (mean ± SD)	1.35 ± 1.74	0.33 ± 0.07
Years after onset (year) (Mean ± SEM)	9.4 ± 0.81	ND
Previous rheumatoid nodules (%/n)	9.43% (10)	0% (0)
Current rheumatoid nodules (%/n)	16% (17)	0% (0)
Previous Raynaud’s phenomenon (%/n)	0.9% (1)	0% (0)
Current Raynaud’s phenomenon (%/n)	1.8% (2)	0% (0)
Previous cutaneous vasculitis (%/n)	0% (0)	0% (0)
Current cutaneous vasculitis (%/n)	0.9% (1)	0% (0)
Bone erosion (%/n)	79.2% (84)	0% (0)

*ND* not determined

### IL-17A serum levels but not Th17 cells are increased in RA patients

**Fig. 1:**
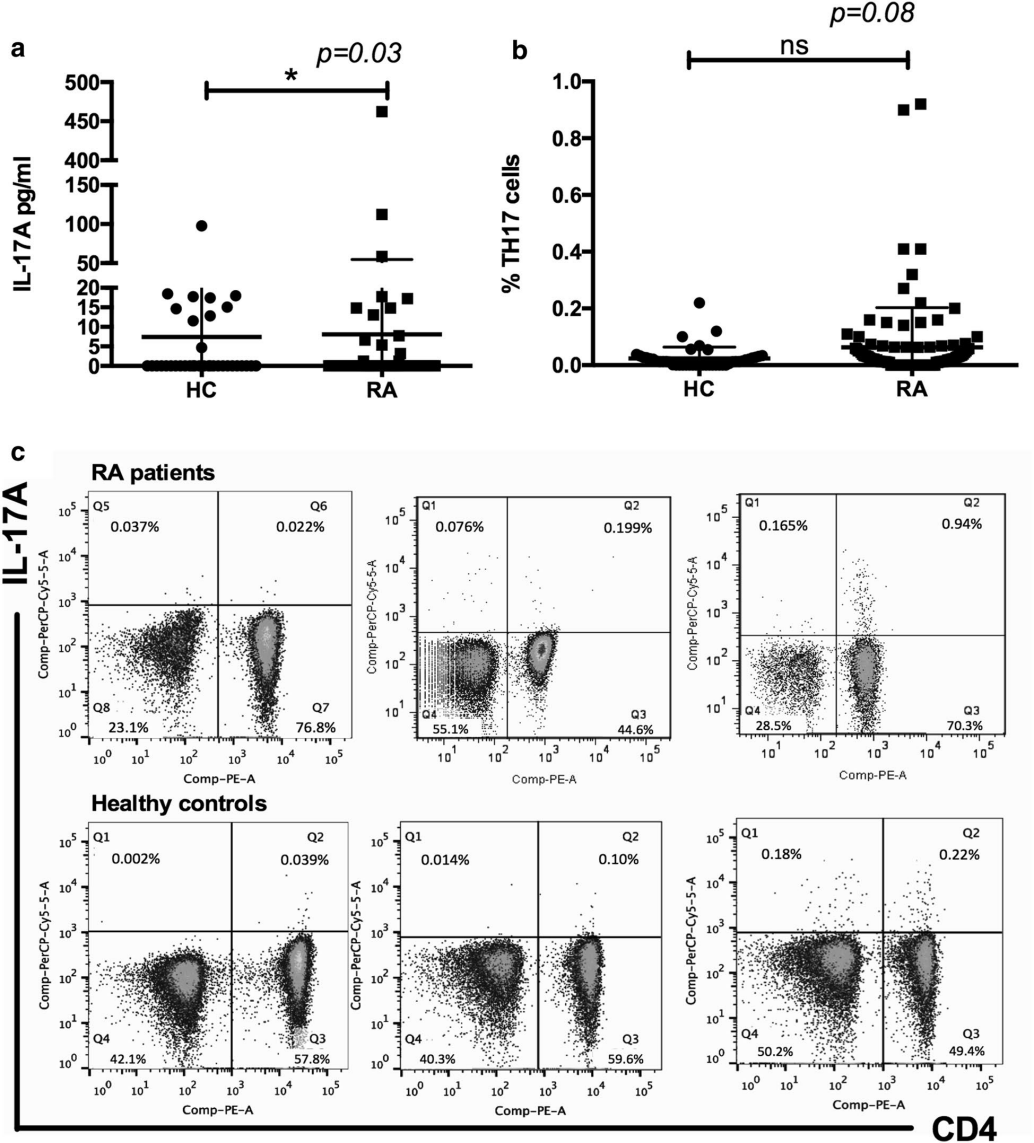
Serum IL-17A levels but not Th17 cell numbers are increased in RA patients. **a** Serum IL-17A was measured by CBA in patients (RA n = 106) and healthy controls (HC n = 56), p = 0.03. **b** ex vivo Th17 cells were identified by flow cytometry from the PBMC fraction, p = 0.08. **c** Three representative dot plots of patients with RA and HCs show IL-17A-producing CD4 T cells. A nonparametric Mann–Whitney test was performed

Increased levels of IL-17A in the serum of patients with RA have been described [5–7]. We measured serum levels of IL-17A in the cohort of RA patients and healthy controls included in this study. We found slightly but statistically significantly increased levels of IL-17A in RA patients compared with those in control subjects (8.0 ± 46.6 pg/ml [0–462.2 pg/ml] vs 7.4 ± 17.2 pg/ml [0–97.68 pg/ml], p = 0.03, Fig. 1a). Because Th17 cells are considered the main producers of IL-17A, we measured the frequency of already polarized Th17 cells in the peripheral blood. This result means that any polyclonal stimuli, such as ionomycin/PMA or anti-CD3/CD28 antibodies, were used to activate T cells in vitro. Therefore, we observed a low frequency of Th17 cells, but there were higher numbers of Th17 cells in RA patients than in healthy controls (0.06% ± 0.14% [0.0–0.92%] in RA patients vs 0.02% ± 0.04% [0.0–0.22%] in controls, *p *= 0.08); however, this difference was not statistically significant (Fig. 1b).

### The frequency of IL-17A+ neutrophils is elevated in patients with RA

**Fig. 2:**
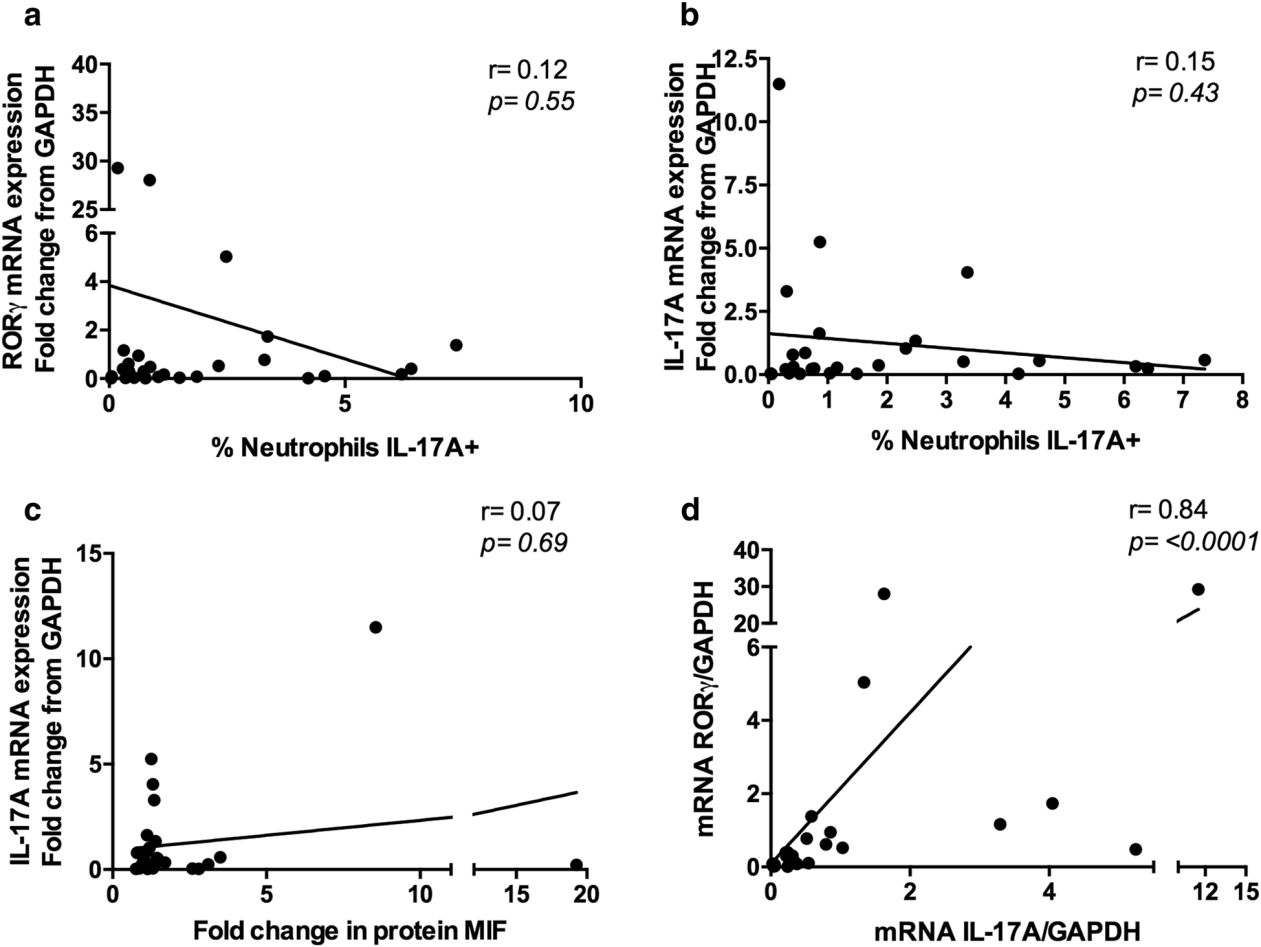
IL-17A mRNA is expressed in neutrophils from RA patients. **a** Correlation plots between RORγ mRNA levels and the frequency of IL-17A + neutrophils from RA patients (n = 27). **b** Correlation plots between IL-17A mRNA levels and the frequency of IL-17A + neutrophils from RA patients (n = 27). **c** Correlation plot between IL-17 mRNA levels and the fold increase in the MFI of IL-17A from RA patients (n = 27). **d** Correlation plots between IL-17 mRNA levels and RORγ mRNA levels from RA patients (n = 27). A Spearman correlation test was performed for these samples

To evaluate the frequency of IL-17A+ neutrophils, we first determined the expression of IL-17A and RORγ mRNA in purified neutrophils from RA patients by means of RT-PCR, both of which were present in neutrophils from RA patients. There was no statistically significant correlation between mRNA levels and the frequency of IL-17A-producing neutrophils (Fig. 2a, b) nor with the mean fluorescence intensity (MFI) of intracellular IL-17A (Fig. 2c). However, a positive correlation (p ≤ 0.0001, r = 0.84) was observed between IL-17A and RORγ mRNA levels (Fig. 2d). Afterward, we studied the frequency of IL-17A+ circulating neutrophils in RA patients and healthy controls. Neutrophil identification was achieved by double staining using anti-CD177 (FITC) and anti-CD66b (PE) antibodies (Additional file 2: Figure S1). IL-17A mRNA expression also correlated with the frequency of IL-17A+ neutrophils in these patients (*p *= 0.008, r = 0.74) (Fig. 2b).

**Fig. 3:**
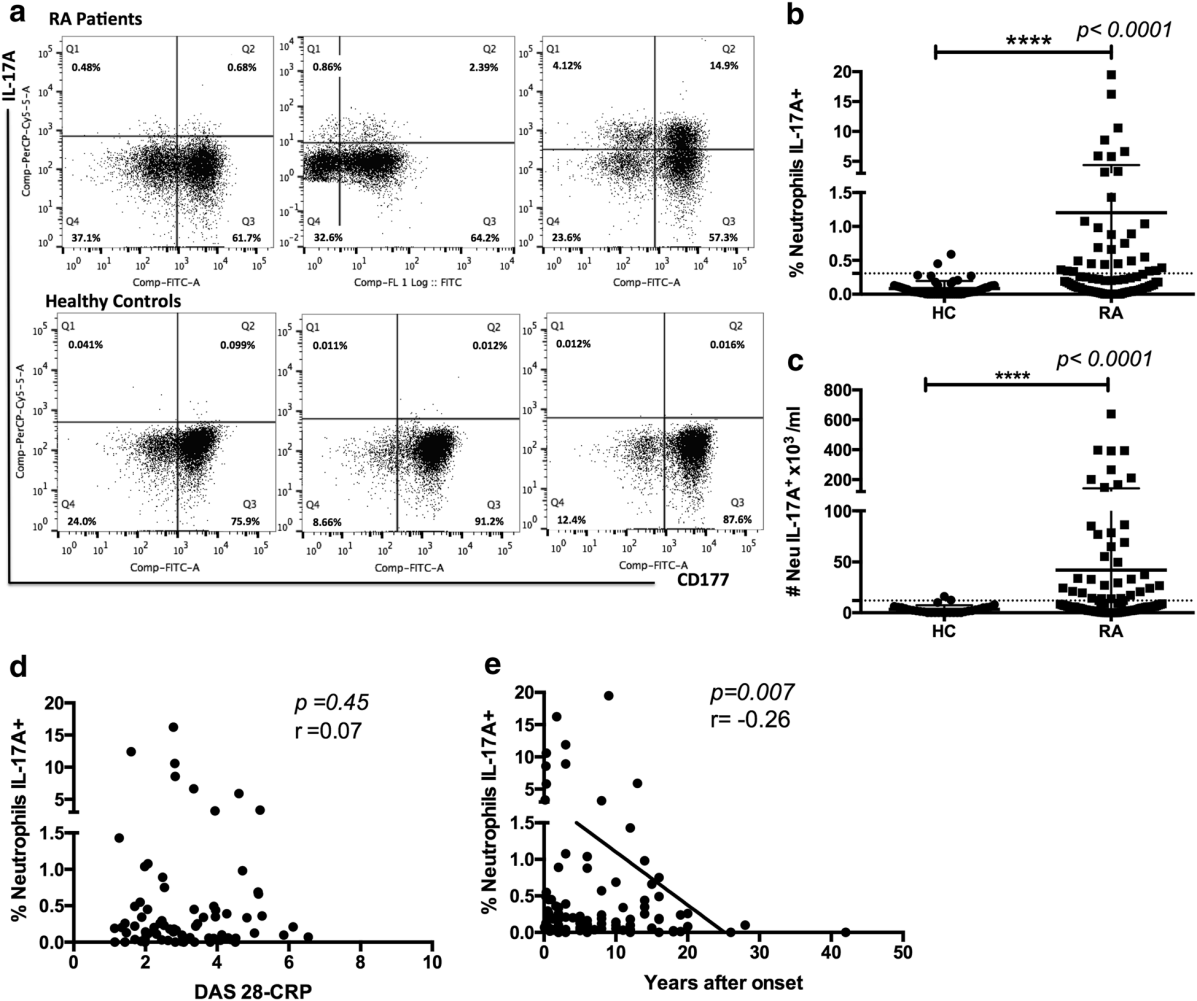
The frequency of IL-17A + neutrophils is elevated in patients with RA. **a** Representative flow cytometry plots for 3 patients and 3 healthy controls. **b** Frequency of IL-17A + neutrophils in patients (n = 106) and healthy controls (56). **c** Absolute numbers of IL-17A-positive neutrophils calculated using neutrophil absolute numbers from the clinical count, using the equation: $\frac{{\# {\text{ total leukocytes }} \times \, \% {\text{ total neutrophils}}}}{100} \times \frac{{\% {\text{ IL}} - 1 7 {\text{A}} + {\text{ neutrophils X 1}}000}}{100}$. The Mann–Whitney U-test was utilized and resulted in a p value < 0.0001. **d** Correlation plot between IL-17A + neutrophils and DAS28-CRP. **e** Correlation graph between IL-17A-producing neutrophils and time after onset of the disease. A Spearman correlation test was performed for these samples. Error bars indicate SD

To analyze whether the frequency of IL17A-producing neutrophils is associated with the disease activity score, a large cohort of RA patients was studied. Figure 3a shows three representative RA patients, one with high (19.02%), one with moderate (3.25%) and one with low frequency (1.16%) IL-17A+ neutrophils. Figure 3a also shows three controls (frequencies of IL-17A+ neutrophils = 0.028%, 0.14% and 0.43%). Regardless of such variability in RA patients, there was a clearly significant difference between both groups (RA mean 1.2 ± 3.18% [0–19.50%] vs. healthy control mean 0.07 ± 0.1% [0–0.59%], *p *< 0.0001, Fig. 3b upper panel). Similar results were observed for absolute numbers of IL-17 + neutrophils (RA mean 42.1 ± 99.4 × 10^3^/ml [0–639.2 × 10^3^/ml] vs. healthy control mean 3.42 ± 4.0 × 10^3^/ml [0–16.14 × 10^3^/ml], *p *< 0.0001) (Fig. 3c). There were no differences in the frequency of IL-17A-producing neutrophils among patients receiving different DMARD combination therapies (data not shown *p *= 0.98).

Based on disease activity, as defined by DAS28-CRP, there was no significant correlation with the frequency of IL-17A+ neutrophils (Fig. 3d, *p *= 0.45, r = 0.07). Moreover, there was no correlation between serum IL-17A and the frequency of IL-17A+ neutrophils.

Neutrophils are innate immune cells that are important in the early stages of the inflammatory response; we observed a positive correlation between the frequency of IL-17A+ neutrophils and the time with clinical diagnosis of RA (Fig. 3f) (*p *= 0.007 *r *= 0.26). Patients with an increased frequency of IL-17A+ neutrophils were those with a relatively recent diagnosis of RA, with a mean time of 3.5 ± 4.24 years from disease onset. In contrast, RA patients negative for IL-17A-producing neutrophils had a mean of 8.2 ± 8.1 years since disease onset.

As IL-6 is necessary for the induction of IL-17A production by neutrophils [28, 29], we measured the serum levels of IL-16, which were increased in RA patients (7.99 ± 16.7 pg/ml in RA vs 0.67 ± 1.18 pg/ml in HC). Additionally, there was a positive correlation between IL-6 serum levels and DAS28-CRP (*p *< 0.0001, r = 0.39), as others have observed [30–32], but IL-6 levels did not correlate with an increased numbers of IL-17A+ neutrophils (data not shown).

### IL-6 and IL-23 induce expression of RORγ but not of IL-17A in neutrophils

**Fig. 4:**
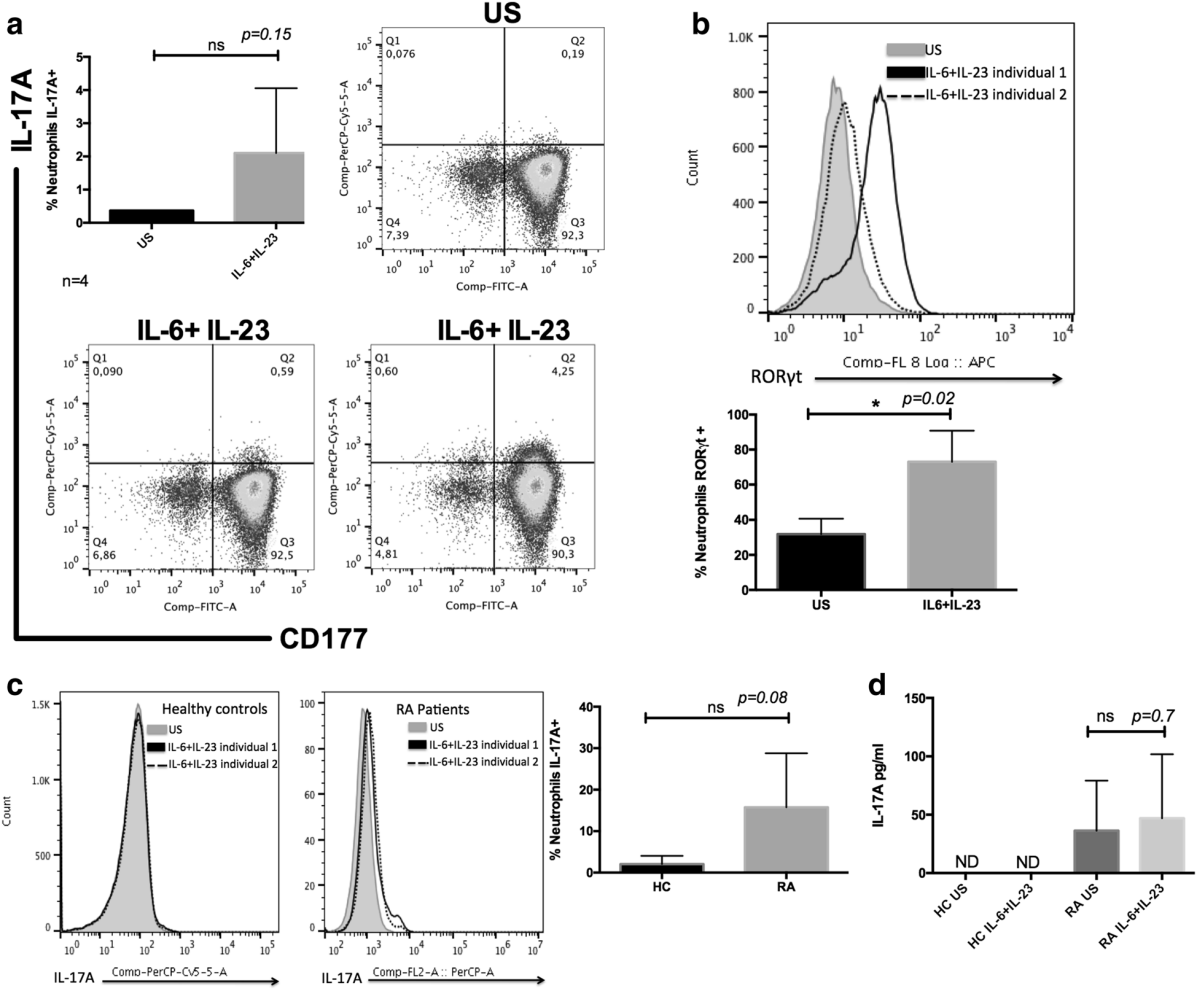
IL-6 and IL-23 are not enough to induce IL-17A production. **a** Neutrophils purified from healthy donors were stimulated with IL-6 and IL-23 for 3 h, and intracellular expression of IL-17A was assessed by flow cytometry. Representative dot plots show IL-17A-producing neutrophils. Unstimulated (US) neutrophils from a healthy donor, and IL-6 + IL-23 stimulates neutrophils from two different healthy donors. The bar graph shows the percentage of IL-17A-producing neutrophils (n = 4). **b** Neutrophils were stimulated as described in **a**, and the frequency of neutrophils expressing RORγ was analyzed by flow cytometry. Representative histograms from unstimulated neutrophils from a healthy donor (gray) and stimulated neutrophils from healthy donors (dashed line and dark lines). The bar graph shows the percentage of RORγ-positive neutrophils (n = 3). **c** Neutrophils isolated from RA patients and HCs were stimulated as in **a** and analyzed for the expression of IL-17A by flow cytometry. Representative histograms of neutrophils from healthy donors and RA patients. Unstimulated neutrophils (gray) and stimulated neutrophils (dashed line and dark lines). The bar graph shows the percentage of IL-17A-producing neutrophils (n = 4). **d** Neutrophils from RA patients and HCs were stimulated as in **a** for 16 h. Afterward, supernatants were collected and analyzed by CBA for IL-17A quantification (n = 4). The Mann–Whitney U-test was utilized and resulted in a p value < 0.0001

As it was reported that IL-6 and IL-23 induce the production of IL-17A and the expression of its master transcription factor RORγ by CD4+ T cells [19, 28, 29], it was of interest to examine whether these cytokines had similar effects on neutrophils. Therefore, purified neutrophils from healthy donors were stimulated with the same concentration of IL-6 and IL-23 as described by Li et al. [18]. The frequency of IL-17A+ neutrophils increased, but it was not statistically significant when compared with that of nonstimulated neutrophils (US = 0.4% ± 0.14%, IL-6 + IL-23 = 2.5% ± 2.1%, *p *= 0.15, Fig. 4a). To analyze whether this effect was similar in neutrophils from patients with RA, neutrophils isolated from RA patients were stimulated with IL-6 and IL-23. We observed an increase in the frequency of RORγ-expressing neutrophils (US 31.8% ± 8.8%, IL-6+ IL-23 72.9% ± 17.7%, *p *= 0.02) (Fig. 4b). Importantly, although there was a trend toward an increased frequency of IL-17A-producing neutrophils in the RA patient group compared with that in the healthy control group, this difference was not statistically significant (HC 2% ± 2, RA 15.7% ± 13%, *p *= 0.08) (Fig. 4c). We wondered if IL-17A could be detected at a later time point of stimulation; for this reason, we measured the cytokine that was being released in the supernatant after 16 h of stimulation. We did not observe IL-17A production by neutrophils from healthy controls (US 36.3 pg/ml ± 42.8 pg/ml; IL-6+ IL-23 46.9 pg/ml ± 54.8 pg/ml, *p *= 0.7) (Fig. 4d).

## Discussion

It has been established that IL-17A is an important cytokine in the pathogenesis of RA. Increased levels of IL-17A have been shown in the synovial fluid of RA patients, and a high concentration of this cytokine is a marker of disease severity. In this study, we observed an increase in the circulating levels of this cytokine. However, a low frequency of Th17 cells ex vivo was observed, with no significant difference from the healthy control group. Nevertheless, Leipe et al. showed that despite the low frequency of Th17 cells observed ex vivo, there was significant difference with healthy controls. This difference from our results might be due to the kind of patients studied; they analyzed patients with very early activity disease (mean disease duration < 3 months), and these patients were treatment-naive patients [33]. In contrast, all RA patients analyzed in the present study were receiving drug treatment (methotrexate, leflunomide or other DMARDs, Additional file 1: Table S1), and the most recently diagnosed patients had a mean of 3.5 years. However, we studied a wide range of patients regarding the mean disease duration since their initial clinical symptoms had occurred 9.4 ± 0.81 years (0–42 years) prior. These criteria were used to improve the understanding of T cell and neutrophil biology in the presence of the inflammatory milieu and immunosuppressive medication. In this context, it has been reported that methotrexate or leflunomide reduces the numbers of Th17 cells in a collagen-induced arthritis mouse model of AR. Thus, it is likely that the medication might have the same effect on human Th17 cells. Consequently, this result suggests that IL-17A might be produced by an alternative source rather than by Th17 cells.

We and other groups have reported neutrophils as a source of IL-17A in asthma patients where an increased frequency of IL-17A+ neutrophils was observed, especially in patients with asthma allergy to fungus [22]. The pathogenic role of IL-17 neutrophils during inflammatory processes in patients with spondyloarthritis has been suggested by observations of this subpopulation of cells found in the facet joints of patients with advanced ankylosing spondylitis [24].

In the present study, we found that peripheral blood IL-17A-producing neutrophil levels were elevated in RA patients, regardless of the level of disease activity. It is likely that these circulating neutrophils might contribute to systemic consequences of RA [34]; by this manner; they might also be involved in the inability to achieve a complete absence of symptoms. However, some of these cells will migrate to the synovial fluid where they might contribute to the local inflammatory response, because, it has been demonstrated that neutrophils CD177+, which co-expressed on their plasma membrane proteinase-3 appear to be selectively recruited for transmigration [35]. IL-17 and RORγ mRNA was detected in neutrophils purified from the peripheral blood of RA patients. The correlation between the levels of both mRNAs suggests that RORγ is a key transcription factor for the development of IL-17A-producing neutrophils. However, the absence of a correlation between IL-17A mRNA levels and IL-17A protein suggests the participation of a regulatory mechanism for both molecules within neutrophils. IL-17A was detected by intracellular staining. Thus, these findings reflect that the production of IL-17A is de novo synthesis and not due to a protein storage model, as has been suggested for neutrophils from psoriatic plaques [36]. Our results also contrast with the data reported by Tammasia et al. because they did not detect the expression of IL-17A mRNA in neutrophils from three patients with psoriasis [37] or if neutrophils were stimulated with cytokines (IFNγ or IL-17A) or TLR ligands (LPS or R848). Remarkably, Tammasi et al. found that the chromatin at the IL-17A locus of resting or stimulated neutrophils displayed a closed conformation. However, they did not rule out that some stimulatory conditions might exist that are able to drive the chromatin modification necessary for IL-17A transcription. Thus, our results suggest that neutrophils of RA patients receive stimulation in vivo that triggers transcription and transduction of the IL-17A gene. Identification of such stimuli was outside the scope of this study. However, it is likely that some of the DAMPS, such as hyaluronan, fibronectin, and collagen, might signal through TLRs to induce the production of this cytokine.

It has been demonstrated that neutrophils from asthmatic patients or from healthy individuals produce increased amounts of IL-17A when stimulated with IL-6, IL-23 and IL-21 [29]. It has also been reported that IL-6 and IL-23 induce the expression of IL-17A mRNA and protein in mouse neutrophils and human neutrophils from healthy individuals [28]. We found high levels of IL-6 in patients with RA, although there was no correlation with the frequency of IL-17A+ neutrophils. We observed, as has been described for Th17 cells, that IL-6 and IL-23 induce the expression of the transcription factor RORγt but only a slight increase in the production of IL-17A. Thus, in addition to cytokines, neutrophils need a second signal that triggers the production and secretion of IL-17A. However, it has been recently published that purified neutrophils from healthy donors did not produce IL-17A when they were stimulated with a broad array of stimuli, including LPS, curdlan or TNFα [37]. We hypothesize, as it was posited before, that perhaps some DAMPS produced during the disease might be involved in the production of this cytokine.

To our knowledge, this is the first time that IL-17A-producing neutrophils have been detected in the peripheral blood of RA patients. However, a prospective study should be conducted to identify whether the increased population of circulating neutrophils expressing IL-17A has a predictive value for relapses or worsening of the disease. Additionally, it will be important to define whether the population of neutrophils are stimulated and expanded in bone marrow and then released into circulation or if they are polarized to produce IL-17 in the periphery when they find the stimuli. Furthermore, the frequency of IL-17A+ neutrophils is higher in patients with recent onset of the disease (less than 3.5 years), whereas patients with more than 8 years of disease had lower frequencies, suggesting that these cells might play an important role during the early phases of RA but are still active during even the very chronic stage of the disease. Therefore, we suggest that peripheral blood IL-17A-producing neutrophils might be involved in the innate immune response that contributes to sustaining the inflamed joints observed in RA patients. In conclusion, RA patients have IL-17A-producing neutrophils that appear to result from endogenous stimulation and that could be an important source of this cytokine at the site of inflammation. Thus, it is important to consider neutrophils as potential therapeutic targets in RA.

## Conclusions

IL-17A-producing neutrophils are increased in RA patients, particularly in recent onset disease patients. This finding suggests that IL-17A-producing neutrophils play an early role in the development of RA.

## Additional files

**Additional file 1: Table S1.** DMARD therapy administrated to patients.

**Additional file 2. Figure S1.** Strategy used for neutrophils identification. A) Neutrophils were identify according to size (forward scatter) and complexity (side scatter) and B) to the expression of CD66b and CD177, an isotype control antibody were used to identify positive to IL-17A.

## Data Availability

Not applicable.

## Abbreviations

CBA: cytometry bead array
CCP: cyclic citrullinate peptide
CRP: C reactive protein
DMARDs: disease-modifying antirheumatic drugs
ESR: erythrocyte sedimentation rate
Th: T helper lymphocytes
TNF: tumor necrosis factor
IFN-γ: interferon gamma
RF: rheumatoid factor
ROS: reactive oxygen species
RORγ: related orphan receptor gamma
PMA: phorbol 12-myristate 13-acetate
TLR: Toll-like receptors

## References

[1] Wright HL, Moots RJ, Edwards SW (2014). The multifactorial role of neutrophils in rheumatoid arthritis. Nat Rev Rheumatol.

[2] Kaneko Y, Takeuchi T (2017). Targeted antibody therapy and relevant novel biomarkers for precision medicine for rheumatoid arthritis. Int Immunol.

[3] Chabaud M, Durand JM, Buchs N, Fossiez F, Page G, Frappart L (1999). Human interleukin-17: a T cell-derived proinflammatory cytokine produced by the rheumatoid synovium. Arthritis Rheum.

[4] Kellner H (2013). Targeting interleukin-17 in patients with active rheumatoid arthritis: rationale and clinical potential. Ther Adv Musculoskelet Dis.

[5] McInnes IB, Schett G (2007). Cytokines in the pathogenesis of rheumatoid arthritis. Nat Rev Immunol.

[6] Kotake S, Udagawa N, Takahashi N, Matsuzaki K, Itoh K, Ishiyama S (1999). IL-17 in synovial fluids from patients with rheumatoid arthritis is a potent stimulator of osteoclastogenesis. J Clin Investig.

[7] Sarkar S, Justa S, Brucks M, Endres J, Fox DA, Zhou X (2014). Interleukin (IL)-17A, F and AF in inflammation: a study in collagen-induced arthritis and rheumatoid arthritis. Clin Exp Immunol.

[8] Katayama M, Ohmura K, Yukawa N, Terao C, Hashimoto M, Yoshifuji H (2013). Neutrophils are essential as a source of IL-17 in the effector phase of arthritis. PloS ONE..

[9] Nirula A, Nilsen J, Klekotka P, Kricorian G, Erondu N, Towne JE (2016). Effect of IL-17 receptor A blockade with brodalumab in inflammatory diseases. Rheumatology..

[10] Kunwar S, Dahal K, Sharma S (2016). Anti-IL-17 therapy in treatment of rheumatoid arthritis: a systematic literature review and meta-analysis of randomized controlled trials. Rheumatol Int.

[11] Pappu R, Ramirez-Carrozzi V, Sambandam A (2011). The interleukin-17 cytokine family: critical players in host defence and inflammatory diseases. Immunology.

[12] Bi Y, Zhou J, Yang H, Wang X, Zhang X, Wang Q (2014). IL-17A produced by neutrophils protects against pneumonic plague through orchestrating IFN-gamma-activated macrophage programming. J Immunol.

[13] Chen F, Cao A, Yao S, Evans-Marin HL, Liu H, Wu W (2016). mTOR mediates IL-23 induction of neutrophil IL-17 and IL-22 production. J Immunol.

[14] Ferretti S, Bonneau O, Dubois GR, Jones CE, Trifilieff A (2003). IL-17, produced by lymphocytes and neutrophils, is necessary for lipopolysaccharide-induced airway neutrophilia: IL-15 as a possible trigger. J Immunol.

[15] Grund LZ, Novaski I, Quesniaux VF, Ryffel B, Lopes-Ferreira M, Lima C (2017). Neutrophils releasing IL-17A into NETs are essential to plasma cell differentiation in inflamed tissue dependent on IL-1R. Autoimmunity..

[16] Karthikeyan RS, Vareechon C, Prajna NV, Dharmalingam K, Pearlman E, Lalitha P (2015). Interleukin 17 expression in peripheral blood neutrophils from fungal keratitis patients and healthy cohorts in southern India. J Infect Dis.

[17] Li L, Huang L, Vergis AL, Ye H, Bajwa A, Narayan V (2010). IL-17 produced by neutrophils regulates IFN-gamma-mediated neutrophil migration in mouse kidney ischemia-reperfusion injury. J Clin Investig.

[18] Li TJ, Jiang YM, Hu YF, Huang L, Yu J, Zhao LY (2017). Interleukin-17-producing neutrophils link inflammatory stimuli to disease progression by promoting angiogenesis in gastric cancer. Clin Cancer Res.

[19] Tan Z, Jiang R, Wang X, Wang Y, Lu L, Liu Q (2013). RORgammat+ IL-17+ neutrophils play a critical role in hepatic ischemia-reperfusion injury. J Mol Cell Biol.

[20] Hueber AJ, Asquith DL, Miller AM, Reilly J, Kerr S, Leipe J (2010). Mast cells express IL-17A in rheumatoid arthritis synovium. J Immunol.

[21] Moran EM, Heydrich R, Ng CT, Saber TP, McCormick J, Sieper J (2011). IL-17A expression is localised to both mononuclear and polymorphonuclear synovial cell infiltrates. PLoS ONE.

[22] Ramirez-Velazquez C, Castillo EC, Guido-Bayardo L, Ortiz-Navarrete V (2013). IL-17-producing peripheral blood CD177+ neutrophils increase in allergic asthmatic subjects. Allergy Asthma Clin Immunol.

[23] Lin AM, Rubin CJ, Khandpur R, Wang JY, Riblett M, Yalavarthi S (2011). Mast cells and neutrophils release IL-17 through extracellular trap formation in psoriasis. J Immunol.

[24] Appel H, Maier R, Wu P, Scheer R, Hempfing A, Kayser R (2011). Analysis of IL-17+ cells in facet joints of patients with spondyloarthritis suggests that the innate immune pathway might be of greater relevance than the Th17-mediated adaptive immune response. Arthritis Res Ther..

[25] Bromley M, Woolley DE (1984). Histopathology of the rheumatoid lesion. Identification of cell types at sites of cartilage erosion. Arthritis Rheum.

[26] Singh JA, Saag KG, Bridges SL, Akl EA, Bannuru RR, Sullivan MC (2016). 2015 American college of rheumatology guideline for the treatment of rheumatoid arthritis. Arthritis Rheumatol.

[27] Combe B, Landewe R, Daien CI, Hua C, Aletaha D, Alvaro-Gracia JM (2017). 2016 update of the EULAR recommendations for the management of early arthritis. Ann Rheum Dis.

[28] Taylor PR, Roy S, Leal SM, Sun Y, Howell SJ, Cobb BA (2014). Activation of neutrophils by autocrine IL-17A-IL-17RC interactions during fungal infection is regulated by IL-6, IL-23, RORgammat and dectin-2. Nat Immunol.

[29] Halwani R, Sultana A, Vazquez-Tello A, Jamhawi A, Al-Masri AA, Al-Muhsen S (2017). Th-17 regulatory cytokines IL-21, IL-23, and IL-6 enhance neutrophil production of IL-17 cytokines during asthma. J Asthma..

[30] Milman N, Karsh J, Booth RA (2010). Correlation of a multi-cytokine panel with clinical disease activity in patients with rheumatoid arthritis. Clin Biochem.

[31] Uson J, Balsa A, Pascual-Salcedo D, Cabezas JA, Gonzalez-Tarrio JM, Martin-Mola E (1997). Soluble interleukin 6 (IL-6) receptor and IL-6 levels in serum and synovial fluid of patients with different arthropathies. J Rheumatol.

[32] Rajaei E, Mowla K, Hayati Q, Ghorbani A, Dargahi-Malamir M, Hesam S (2019). Evaluating the relationship between serum level of interleukin-6 and rheumatoid arthritis severity and disease activity. Curr Rheumatol Rev.

[33] Leipe J, Grunke M, Dechant C, Reindl C, Kerzendorf U, Schulze-Koops H (2010). Role of Th17 cells in human autoimmune arthritis. Arthritis Rheum.

[34] McInnes IB, Schett G (2011). The pathogenesis of rheumatoid arthritis. N Engl J Med.

[35] Kuckleburg CJ, Tilkens SB, Santoso S, Newman PJ (2012). Proteinase 3 contributes to transendothelial migration of NB1-positive neutrophils. J Immunol.

[36] Reich K, Papp KA, Matheson RT, Tu JH, Bissonnette R, Bourcier M (2015). Evidence that a neutrophil-keratinocyte crosstalk is an early target of IL-17A inhibition in psoriasis. Exp Dermatol.

[37] Tamassia N, Arruda-Silva F, Calzetti F, Lonardi S, Gasperini S, Gardiman E (2018). A reappraisal on the potential ability of human neutrophils to express and produce IL-17 family members in vitro: failure to reproducibly detect it. Front Immunol.

