# Supplementary material for: Endogenous stimulation is responsible for the high frequency of IL-17A-producing neutrophils in patients with rheumatoid arthritis
Source: Allergy Asthma Clin Immunol. 2019 Aug 1;15:44. doi: 10.1186/s13223-019-0359-9 (PMC6676628; doi:10.1186/s13223-019-0359-9)
Supplement: Supplementary file 1 — Additional file 1: Table S1. DMARD therapy administrated to patients. [file 13223_2019_359_MOESM1_ESM.docx]

| **Synthetic DAMRD** | **Percentage of patients receiving medication %(n)** | **Prednisone** |
| --- | --- | --- |
| MTX | 27.1% (28) | 2.9% (3) |
| LEF | 2.9% (3) |  |
| HCQ | 3.8% (4) |  |
| SSZ | 7.6% (8) | 3.8%(4) |
| MTX + SSZ | 13.5% (16) |  |
| MTX + HCQ | 21.6% (23) | 5.6% (6) |
| MTX + LEF | 2.9% (3) |  |
| HCQ + SSZ | 3.8% (4) | 0.9% (1) |
| SSZ + LEF | 3.8% (4) |  |
| MTX + HCQ + SSZ | 3.8% (4) | 0.9% (1) |
| HCQ + SSZ + LEF | 2.9% (3) |  |
| MTX + SSZ + LEF | 2.8% (3) | 0.9% (1) |
| MTX+ HCQ + LEF | 1.94% (2) | 0.9% (1) |

**Table S1: DMARD therapy administrated to patients**

MTX= Methotrexate; LEF=Leflunomide; HCQ=Hydroxychloroquine; SSZ=Sulfasalazine

All patients were receiving low dose of glucocorticoid ≤10mg/day.
