# Supplementary figures and images for: Endogenous stimulation is responsible for the high frequency of IL-17A-producing neutrophils in patients with rheumatoid arthritis
Source: Allergy Asthma Clin Immunol. 2019 Aug 1;15:44. doi: 10.1186/s13223-019-0359-9 (PMC6676628; doi:10.1186/s13223-019-0359-9)

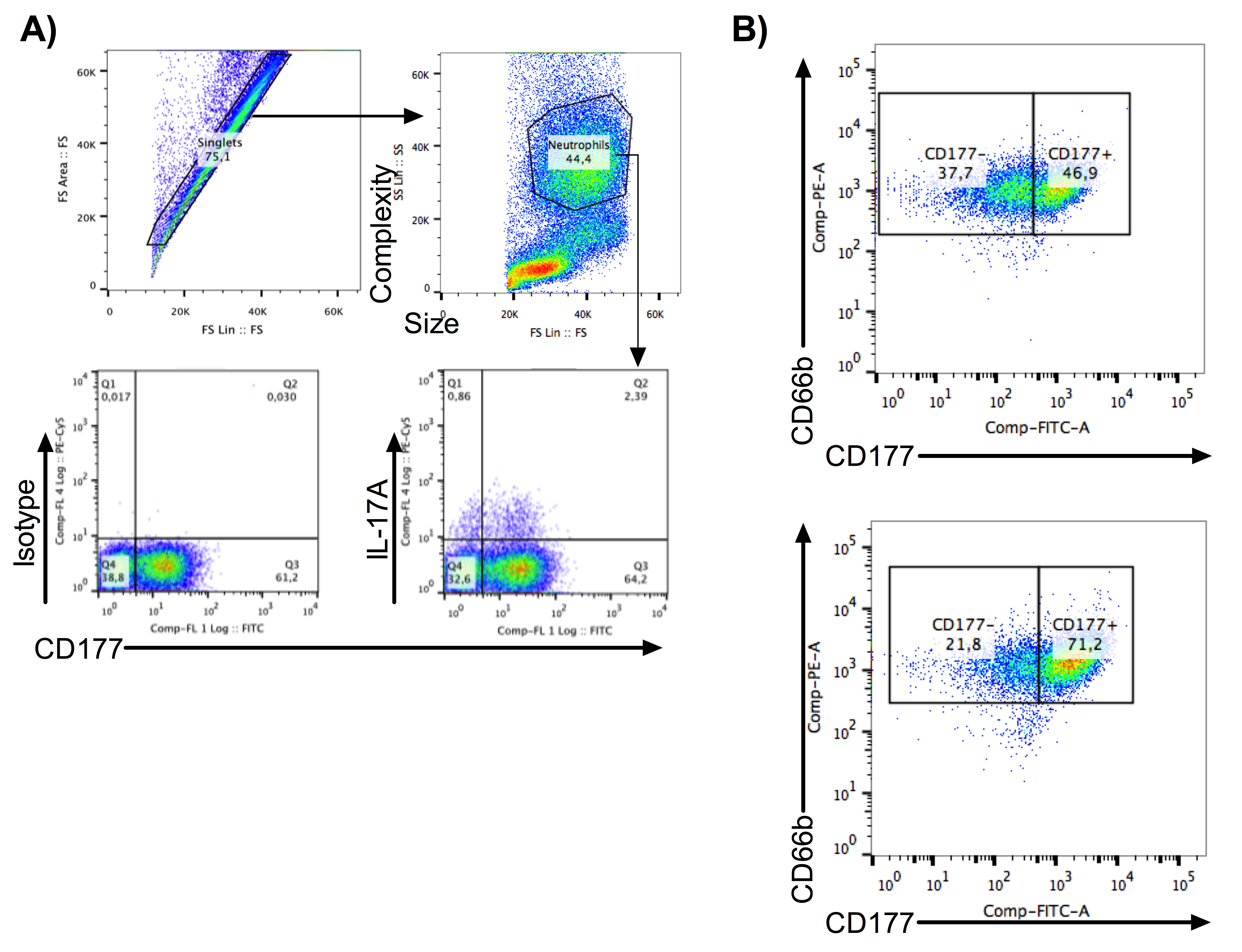

Supplement: Supplementary file 2 — Additional file 2. Figure S1. Strategy used for neutrophils identification. A) Neutrophils were identify according to size (forward scatter) and complexity (side scatter) and B) to the expression of CD66b and CD177, an isotype control antibody were used to identify positive to IL-17A. [file 13223_2019_359_MOESM2_ESM.tiff]
